# Organic Control Strategies for Use in IPM of Invertebrate Pests in Apple and Pear Orchards

**DOI:** 10.3390/insects12121106

**Published:** 2021-12-11

**Authors:** Bethan Shaw, Csaba Nagy, Michelle T. Fountain

**Affiliations:** 1 NIAB EMR, East Malling, Kent ME19 6BJ, UK; michelle.fountain@niab.com; 2 Research Centre for Fruit Growing, Institute of Horticultural Science, Hungarian University of Agriculture and Life Sciences, 2100 Budapest, Hungary; bigjabba@gmail.com

**Keywords:** biological control, codling moth, cultural control, fruit, physical control

## Abstract

**Simple Summary:**

Reductions in the numbers of chemical plant protection products that are approved and a move towards organic production has changed the way growers produce fruit in Europe. This is likely the result of public pressure and the need for less reliance on broad-spectrum interventions. This review summarises organic approaches that could be adopted as part of integrated pest management strategies in apple and pear orchards. It focuses on control methods to target key and emerging European insect pests through biological, cultural, and physical control strategies.

**Abstract:**

Growers of organic tree fruit face challenges in controlling some pests more easily suppressed by broad-spectrum insecticides in conventionally managed orchards. In recent decades, there has been a move towards organically growing varieties normally reliant on synthetic chemical pesticides (e.g., Gala), often to meet retailer/consumer demands. This inevitably makes crop protection in organic orchards more challenging, as modern varieties can be less tolerant to pests. In addition, there have been substantial reductions in plant protection product (PPP) approvals, resulting in fewer chemical options available for integrated pest management (IPM)-maintained orchards. Conversely, the organic management of fruit tree pests involves many practices that could be successfully implemented in conventionally grown crops, but which are currently not. These practices could also be more widely used in IPM-maintained orchards, alleviating the reliance on broad-spectrum PPP. In this review, we evaluate organic practices, with a focus on those that could be incorporated into conventional apple and pear production. The topics cover cultural control, biological control, physical and pest modifications. While the pests discussed mainly affect European species, many of the methods could be used to target other global pests for more environmentally sustainable practices.

## 1. Introduction

The incentive for growers to adopt organic methods has been driven by consumer attitudes and an increased awareness of the impacts that some agricultural practices have on the environment [1]. However, organic agriculture typically produces 8–25% lower yields than conventional production [2], and in apple production, a ~48% reduction in yield has been recorded compared with conventional and integrated pest management (IPM) orchards [3]. There are also concerns that fully organic systems alone will not meet the increasing food demand for our expanding populations [4,5,6,7]. Whilst there are positive impacts associated with organic agriculture, such as increased biodiversity [8,9], there is an economic and societal requirement for a balance between high yields, reduced waste and land use [10]. Organic production, as of 2017, covers 69.8 million hectares worldwide, but only 1.6% of temperate fruit is grown organically [11]. Apples comprised 40% of organic temperate fruit production in 2017, the largest proportion of all temperate fruit, with pears accounting for 10% [11]. In 2018, the UK market production of apples (dessert and culinary) and pears (dessert) was 300.6 and 26.6 thousand tonnes, imports were 372 and 120 thousand tonnes, and exports were 22 and 2 thousand tonnes, respectively. In 2020, dessert apple production was valued at approximately GBP 158 million from over 6000 hectares of land in the UK (Table 1).

An increase in the area of apples and pears grown, coupled with the recent and rapid reduction in the number of plant protection product (PPP) approvals, also threatens the pome fruit industry. The loss of organophosphates and some pyrethroid and neonicotinoid foliar sprays over recent years has resulted in a resurgence of key pests and diseases in European apple and pear orchards. There have been additional yield losses due to fruit damage caused by sporadic pests that would have been suppressed by these broad-spectrum products [12], e.g., damage by the forest bug *Pentatoma rufipes* (L.) [13]. Impending PPP withdrawals, such as thiacloprid at the end of 2021, are expected to result in increases in intermittent pests such as weevils, capsids, and aphids. In addition, there are some invasive species that are likely to be a future risk to the industry, and control options are needed to prevent yield loss, for example the brown marmorated stink bug (BMSB) *Halyomorpha halys* (Stål) [14]. As more broad-spectrum PPPs lose approval [15], growers need to adapt and be receptive to alternative methods for fruit pest control.

Pest pressure in organic orchards can result in high yield losses, which may be difficult to estimate, although direct damage to fruit caused by pests such as the codling moth, *Cydia pomonella* (L.), can be quantified at harvest. However, not all damage is easily identifiable, and true losses may be much higher than estimated [16]. Much pest damage is indirect—for example, apple fruit weevils, *Tatianaerhynchites aequatus* (L.), sever the stem of developing apple fruitlets, causing them to fall to the ground after egg laying, coinciding with June-drop in the UK. Pear suckers, *Cacopsylla pyri* (L.), reduce plant health by feeding on leaves and overwintering buds, causing subsequent yield reductions.

This review focuses on organic strategies for achieving effective pest control in apple and pear orchards, with specific emphasis on European invertebrate pests. The control strategies discussed can be adopted within IPM programs and, in some cases, substitute or complement PPP-based pest control. While there are several synthetic organically approved products, biostimulants, and physically acting compounds used in IPM and organic fruit production (e.g., spinosad, neem, FLiPPER, etc.), they are not discussed in this review. This review focuses on alternative methods to foliar products, with the exception of biopesticides including viruses, entomopathogenic nematodes and fungi.

## 2. Cultural Control

Cultural control prevents or discourages pest populations by optimising growing parameters and improving plant health and husbandry [17], and can be both crop- and/or surrounding habitat-focused. Many techniques are implemented prior to planting and are applicable in both organic and IPM orchards.

### 2.1. Soil Health and Properties

Soil characteristics and health can dictate the occurrence of pests within a crop and varies between farm and location. Prior to establishing an orchard or new planting, it is beneficial to identify the soil type and its properties to give an indication of qualities such as drainage, soil composition and soil quality. These factors may impact fruit production after orchard establishment [18] and determine the most appropriate pre-planting soil amendments. Soil health is vital in organic and IPM orchard pest management; however, above- and below-ground management strategies are rarely combined in ‘ecologically based pest management’ approaches [19]. Higher soil fertility and organic matter content have been linked to lower pest pressure, but excessive nutrient levels have adverse effects by prompting excessive new tree growth which can be colonised by aphids [20]. In pears, high nitrogen levels result in higher numbers of pear suckers, *Cacopsylla pyricola* (Foerster), as a result of more foliar growth [21]. These examples demonstrate the balance between improving soil properties and avoiding favouring pest insects.

### 2.2. Cover Crops

Methods used to improve soil fertility for pest and disease resilience in trees include cover cropping pre-planting [22]. Cover crops protect soil and amend soil properties including nitrogen and organic matter content. Wildflower mixes could be considered for the long-term management of orchards to provide habitats for beneficial insects including pollinators [23,24]. Cover crops modify nutrient levels in soil and healthy soils result in healthy plants, more tolerant to pests and diseases than those grown in poor soils [20,22]. However, not all cover crops are beneficial; some increase soil fertility above optimum levels [25]. In an organic pear orchard sown with the legume hairy vetch, *Vicia villosa* Roth, the K content was higher than control plots, but plots planted with barley, *Hordeum vulgare* L., or rye, *Secale cereale* L., had lower K than the uncovered control plots [26]. Pome fruit varieties also have different nutritional needs, which should be considered before planting cover crops. For example, cv. Comice pears are frequently deficient in Mg [27], and so barley and hairy vetch would be incompatible, as lower Mg levels occurred in plots covered with these species [26]. Common vetch, *Vicia sativa* L., as a cover crop caused a sharp increase in soil nitrates in the spring and increased soil organic matter following the decomposition of cuttings [28]. During this time, there was also an increase in the total number of soil nematodes and, although no predatory species were collected, high nematode numbers indicate good carbon flow within an orchard soil [29]. These researchers attributed increased nematode numbers to increasing soil organic matter from the cuttings.

Many non-pest herbivorous invertebrates utilize cover crops providing predators and parasitoids with alternative food sources [30]. Marking natural enemies with egg albumen protein, and analysing by monoclonal antibody techniques, it was possible to demonstrate that beneficial insects moved between cover crops and pear trees. In field trials, 17–29% of predators collected on pear trees were marked, indicating that they had either migrated from, or previously visited, the immune marker-treated cover crops [31]. Marked predators included species of Heteroptera (*Anthocoris* sp.), Coccinellidae (ladybirds), Chrysopidae (lacewings), and Araneae (spiders), all common natural enemies in apple and pear orchards. This topic is described in more detail in the ‘Natural enemies’ Section 3.1 of this review.

### 2.3. Variety

Varietal choice is a primary consideration in preventing pest damage to tree fruit. However, cultivar plantings are often driven by markets, retailer demands and producer organization variety requirements. A reliance on varieties that are high yielding but more susceptible to pests is a barrier to reducing the reliance on PPP. Certain apple scion varieties, such as Florina and Prima, are less susceptible to aphids, including rosy apple aphid, *Dysaphis plantaginea* (Passerini) [32], and green-apple aphid, *Aphis pomi* De Geer [33]. Rosy apple aphid resistance is linked to the presence of hydroxycinnamic acids, common in cider apple varieties. Hydroxycinnamic acids protect fruit skin from UV light [34]. As such, plant breeding that incorporates crosses between varieties that are high in hydroxycinnamic acids could increase the availability of tolerant cultivars.

Marker-assisted breeding has identified genetic markers associated with resistance to rosy apple aphid [35], green-apple aphid [36], and leaf-curling aphid, *Dysaphis devecta* (Walker) [37]. These markers should be targeted in the selection of future cultivars. The woolly apple aphid, *Eriosoma lanigerum* (Hausmann), is controlled by varietal resistance in many New Zealand-bred cultivars, which are developed for a temperate climate, and may be appropriate for other temperate growing regions. These varieties include Geneva, Willie Sharp, and Korichnoe Polosatoje G01-104 [38].

In pears, pear sucker (*C. pyri* and *C. pyricola*)-resistant varieties can be selected [39,40]. The profile of polyphenolic secondary metabolites within pear leaves has been associated with pear sucker resistance. Increases in these compounds are linked to the plant’s self-defence mechanism against pathogens and UV, which in turn increases fitness [41]. Microsatellite markers associated with resistance to the pear-bedstraw aphid, *Dysaphis pyri* (Boyer de Fonscolombe), and QTL markers associated with the pear sawfly (*Caliroa cerasi* (L.)) and pear blister mite (*Eriophyes pyri* (Pagenstecher)) resistance have also been identified in European pear varieties and can be identified early in the screening of varietal development [42,43].

Apple rootstocks, resistant to woolly apple aphid, such as ‘Northern spy’ (developed at East Malling Research, Kent, UK) [44], are also available, but there is little to indicate that they are widely used for this purpose [45]. At the time of writing, there were no conclusive reports of pear rootstocks that promote pest resistance.

Classical breeding of new apple and pear varieties takes decades, but the integration of marker-assisted breeding techniques could reduce the time from concept to commercialisation while also promoting resistance to key pests and diseases [42,46,47,48,49]. However, marker-assisted fruit-breeding is underexploited, often attributed to cost and the need for expertise [49].

Cultivar phenology also impacts the susceptibility of fruit trees to insect pests by avoiding synchrony of vulnerable stages with the emergence or arrival of pests [50]. In apples, later developing, susceptible varieties have fewer rosy apple aphids, as bud burst occurs after egg hatch and neonates cannot feed [51]. However, in pears, a preference for more advanced stages of leaf emergence was demonstrated for egg-laying winter morph pear suckers, even when susceptible varieties were available. Whilst there are pear varieties resistant to summer morph pear suckers, winter morph insects are more influenced by tree phenology than variety [52]. Fruit growers can use knowledge of pests prevalent on their farms to select cultivars with known resistance and phenology to reduce the need for PPP control measures.

### 2.4. Canopy Maintenance

**Figure 1:**
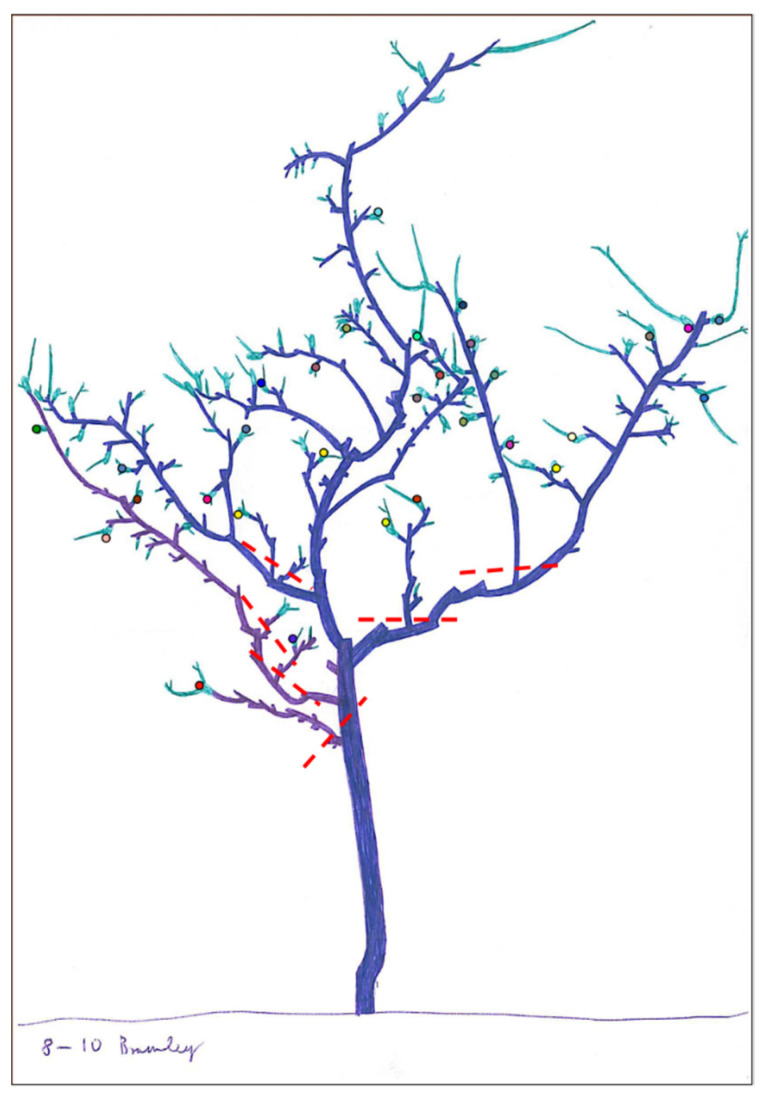
Graphic of real apple tree including shoots removed to reduce rosy apple aphid, *Dysaphis plantaginea*, colonies and disrupt the ant–aphid mutualistic relationship. Red dashed lines indicate areas of growth to be removed. Coloured circles display aphid fundatrices occurring at different times. Illustration by C. Nagy.

Pruning changes environmental conditions in the tree canopy, such as humidity, temperature, airflow and light penetration [53]. In addition to rootstock selection, pruning, nutrient inputs and tree canopy architecture can be controlled through genetic manipulation. Key genes involved with branching and growth can be used in the marker-assisted breeding of future varieties [54]. Training fruit trees as they grow reduced pest and disease pressure in French orchards [55]. For example, centrifugal training of pear and apple promoted a reduction in aphids and scab incidence, attributed to the ability of predators to more easily access pests and a reduction in disease-promoting humidity [56,57]. Growers can combine methods such as pruning and applications of nitrogen to reduce excessive growth, which promote aphid damage [58]. In young orchards, rosy apple aphid colonies can be controlled by the removal of curled leaves, which harbour the fundatrix (founding) aphid during blossom. This method does not require skilled labour and has been found to be extremely effective, although time consuming (C. Nagy, unpublished). Aphid colonies are tended by ants (*Lasius niger* L.) as part of a mutualistic relationship. Ants defend aphid colonies against predators in exchange for honeydew secreted by the aphids, although this relationship is not observed in all aphid species [59]. Colonies that infest the lower and middle tree shoots are located earlier in the season and are more accessible to ants compared to colonies on peripheral shoots (C. Nagy, unpublished). In older orchards, pruning excess growth (the suckers) around the central tree zone prevents aphid colonies from establishing, leaving colonies in the tree periphery where they are more accessible to aerial predators (see natural enemies’ Section 3.1 for more detail). Figure 1 shows the central growth that should be removed to promote rosy apple aphid control. Using this pruning technique, rosy apple aphid damage to the foliage was reduced by 50% compared to an unpruned control (C. Nagy, unpublished).

There is little literature on apple and pear canopy management for the reduction in the populations of other types of pests, but canopy management can increase fruit yield. Apple trees are normally managed to have less dense canopies to improve light and air flow for higher fruit yields and better fruit colouration [60,61], but opportunities for pest insects to hide from larger predators is also reduced. Birds, predominantly Paridae, predate a range of pests in apple and pear orchards [62], particularly caterpillars during the bird nesting season, and are linked to a reduction in pest occurrence and an increase in crop yield [63,64,65,66,67]. Growers could further enhance numbers of Paridae on their farms by providing appropriate nesting habitat and nest boxes [65,67]. While some bird species can cause direct damage fruit, mainly blackbirds *Turdus merula* (L.) and starlings *Sturnus vulgaris* (L.), they generally attack near-ripening fruit [68] and can be deterred with bird scarers or netting. Canopy thinning also aids visual inspections by crop walkers and agronomists, resulting in the quicker detection of pests and timely control. In addition, the coverage of plant protection products such as entomopathogenic viruses and bio-protectants, is improved by good canopy management [69,70].

Labour costs associated with accurate pruning might be prohibitive, but there are opportunities for canopy management from spring through early summer between more time-specific tasks.

## 3. Biological Control

### 3.1. Natural Enemies

Unsprayed fruit trees support a large fauna of >2000 arthropod species, which include pest, beneficial and benign invertebrates [71]. Some insect pests have become more prevalent in orchards due to the adverse effects of PPP on their natural enemies [72,73,74]. Pear sucker prevalence has increased over the past 30 years through insecticide resistance and repeated applications of insecticides toxic to their natural enemies [75]. However, effective control is achievable in orchards that support and promote a healthy and diverse natural enemy network [76,77]. For many predators and parasitoids, a diverse habitat is required to support their full lifecycle. Improved habitat can be facilitated through the planting of pollen, nectar, and structure rich ground cover either within or around the orchard perimeter. Conservation biological control should be tailored to encourage beneficial insects whilst minimising the build-up of other pests and diseases; this is discussed in more detail in Section 3.2. Growers should encourage a wide range of generalist and species-specific natural enemies for a broad range of pests. Natural enemy populations fluctuate throughout the fruit growing season, but there are many naturally occurring generalist predators that suppress pest populations at different times of the year [78].

The pirate bug, *Anthocoris nemoralis* (Fabricius), suppresses pear sucker populations in orchards when chemical controls are removed [79]. *A. nemoralis* and *Anthocoris nemorum* (L.) are the most widely occurring predatory Heteroptera in pear and apple orchards, respectively [80]. Anthocorids are generalist predators of fruit tree red spider mites, *Panonychus ulmi* (Koch), and numerous aphid species [81]. Floral resources, including weeds, sustain anthocorid populations when prey within the orchard is scarce [82]. Non-crop plants encourage anthocorids by supporting non-pest herbivores which act as alternative prey until pest numbers in orchards have begun to build-up. For example, traditional hedgerows which also act as windbreaks around orchards support anthocorids and also benefit other beneficial insects including earwigs, *Forficula auricularia* L. [83]. Goat and grey willow (*Salix caprea* L. and *S. cinerea* L.), hawthorn (*Crataegus monogyna* Jacq.), and nettle (*Urtica dioica* L.) host anthocorids early in the growing season and can be utilised for pear sucker control [84]. Hedgerows have the added benefits of “providing water quality improvement, flood risk reduction, soil loss reduction (erosion), crop water availability, crop pest reduction, crop pollination improvement, shelter provision (crops and livestock), climate change mitigation and urban air quality” [85]. Unbroken, they act as vegetation pathways enabling the movement of pollinators and natural enemies across a landscape [86]. Growers can encourage anthocorids into cropping areas by planting and enhancing the wild hosts of anthocorids in the vicinity of apple and pear orchards. This will promote a more fluid movement of predators between wild and cultivated plants, reducing the lag in predator establishment and pest suppression [87]. However, more work is needed to tailor hedgerows for other pest control and conservation biocontrol. The augmented release of anthocorids from commercial biocontrol companies can also speed up pear sucker control in the spring when naturally occurring anthocorid numbers are low.

Earwigs are generalist predators of pests in apple and pear orchards. Unusually for insects, they care for their brood by tending to the eggs and nymphs in nests [88]. They predate on a range of pests, including aphids, midge larvae, moth larvae [89], and scale pests [90]. In cases where sticky bands were applied to the trunks of apple trees to prevent woolly apple aphid movement, researchers observed an increase in aphid colony size because the banding inadvertently prevented earwigs predating the aphid colonies [45]. A similar bioassay found that ‘Tanglefoot insect exclusion bands’ increased the woolly apple aphid infestation of new apple shoots by 20–25% in comparison to controls in which earwigs could access colonies [91].

Earwigs are also sensitive to many PPPs [92,93], sometimes with sublethal effects difficult to detect in orchards [93]. In soft fruit production, earwigs are regarded as a pest due to their omnivorous diet [89], but do not cause direct damage to apples [94]. Earwigs are nocturnal foragers and spend the daylight hours hiding in crevices in bark or introduced structures that provide refuge, such as supporting canes and tree stakes. More recently, a commercial refuge has been made available to the UK market, ‘Wignest’ (Russell IPM), which provides shelter and a food attractant. Rolled-up corrugated cardboard within a bottomless plastic drinks bottle can provide a simplified refuge and can be supplemented with dried cat food when prey is scarce [95]. These commercial or homemade-equivalent devices are particularly beneficial in young orchards where trees have not yet formed naturally occurring shelters. In orchard trees with more than six earwigs per refuge, woolly apple aphid infestation never exceeded five small colonies per tree [96]. In organic apple orchards, releases of earwigs were performed in aphid-infected trees, resulting in a reduction in aphid numbers from >500 to 50 per tree within three weeks [97]. This greatly contrasted with the 3000 and 2000 aphids per tree in the earwig-free and control plots, respectively. In these orchards, five to six earwigs were released per tree and provided with day refuges within the trees and a straw floor covering to provide shelter for the ground-dwelling nymphs [97].

Hoverflies are a common visitor to many crops, and their importance in providing economic, environmental and ecological services is widely recognised [98,99]. A range of hoverfly species occur in apple and pear orchards and contribute to aphid control, through larval feeding in addition to pollination from adults hoverflies [100]. Hoverflies use a combination of chemical volatiles to locate prey, some of which are emitted by the aphid and others from the attacked plant [101]. Encouraging flora, in particular pollen- and nectar-rich species, and alternative food sources for predacious larvae is vital to both prolong the life and build up populations of hoverflies local to crops. The provision of alyssum, *Lobularia maritima* (L.), can improve hoverfly diversity and subsequent reduction in aphids on crops [102,103]. *Episyrphus balteatus* (De Geer) is a specialist predator of aphids and an important pollinator of fruit crops [104,105]. The larvae are voracious predators capable of consuming large numbers of aphids even at low temperatures (15 °C) [106,107], hence may be effective predators in the spring when pear and apple trees are in flower.

Nettle (*Urtica*) is a host to the nettle aphid, *Microlophium carnosum* (Buckton), which acts as a ‘reservoir’ for many natural enemies [108]. Populations of nettle aphids increase from late April [108], and provide a resource for biocontrol agents to establish during the period in which cultural control practices delay the expansion of aphid colonies on trees. Over 100 insect species have been identified on the common nettle, *U. dioica* L., including ladybirds, lacewings, hoverflies and parasitoids [109]. Ladybirds are generalist predators common in both commercial crops and wild habitats and are less prevalent in conventional than in organic apple [110]. Ladybird larval predation of pests typically begins just before apple flowering, which coincides with PPP applications, resulting in the disruption of this predator [110].

The control of aphids by a range of predators and parasitoids can be disrupted by colonies being tended by ants in a mutualistic relationship. In the UK the common black ant, *Lasius niger* (L.), typically tends colonies of rosy apple aphids and green apple aphids, and has been observed defending aphids from parasitic wasps [71]. In field experiments in both the UK and Hungary, the exclusion of ants from aphid colonies and the provision of ants with sugar feeders resulted in an increase in predation from naturally occurring predators, including hoverflies [111,112,113]. Ants prevented from reaching the aphids by exclusion bands or supplemented with sugar feeders did not defend aphid colonies, and this enabled aerial predators (e.g., hoverflies and ladybirds) to access the aphids [114]. Aphidiinae parasitic wasp species, in particular, are able to exploit undefended aphid colonies but are not sufficient on their own to prevent economic damage [75].

For some predatory species, growers can support populations through the management of wild hosts. Common nettles could be left uncut and native hedgerow species planted instead of current Italian Alder, *Alnus cordata* (Loisel.) Duby. Currently, in the UK, growers can claim GBP 11 per meter under grant number BN11: Planting new hedges within the Countryside Stewardship grants [115]. The deployment of commercial or homemade refuges for earwigs would require initial financial and labour inputs but could be in place for several seasons, building up earwig populations in fruit trees, making it cost effective in the long term.

### 3.2. Introduced/Augmented Biological Control

Although historically applied in glasshouses or protected cropping [78], the augmented release of some predatory mites can be performed outdoors. Large numbers can be introduced either as a preventative or curative treatment, but temperature requirements and the timing of releases should be considered. In glasshouses, predatory mite populations establish quickly and can be implemented for curative treatments. In outdoor orchards, predatory mite populations take longer to increase, and so deploying them before pest outbreaks may mitigate the lag often observed between introduction and predation. Predatory mites can be introduced to suppress a wide number of pest mites, including the two spot spider mite, *Tetranychus urticae* Koch [116], fruit tree red spider mite, *P. ulmi* [117], apple rust mite, *Aculus schlechtendali* (Nalepa) [118], and pear rust mite, *Epitrimerus pyri* (Nalepa) [119].

The temperature range also influences the application timing of biological control agents and the recommendations of manufacturers should be heeded for optimum efficacy. *Phytoseiulus persimilis* Athias-Henriot, used to suppress the two-spot spider mite, requires a temperature range of 15–28 °C to successfully establish and actively predate [120]. *P. persimilis* females consume, on average, 370 two-spot spider mite eggs in their life time, with 320 of these being while they themselves are egg laying within this temperature range [121]. *Amblyseius andersoni* (Chant) can be introduced to control two-spot spider mites and fruit tree red spider mites and has a much wider temperature range than *P. persimilis*. *A. andersoni* is active at 6–40 °C [122] and consumes more adult spider mites than *P. persimilis*. However, the former has a lower rate of population increase [123]. *A. andersoni* are attracted to apple branches infested with fruit tree red spider mite, possibly as a response to the emission rates of volatile organic compounds from the trees influenced by pest pressure [124]. These factors indicate that plant volatile cues could be combined with pest volatiles to encourage or attract predatory mites to specific areas of a crop.

Predatory mites are used widely in protected crops, but their use in unprotected orchards appears to be minimal. This may be due to the costs of implementation, which reflects the high costs associated with the mass production of these predators [125]. The cost coupled with the delay in noticeable impact on pest populations is likely to be the cause of the lack of uptake by growers. However, growers can encourage natural establishment of predatory mites. Orchard leaf litter, the growing tips from other crops, such as strawberry or vines with a high predator population density can be transferred during the autumn [126,127]. *Typhlodromus pyri* Scheuten, the main predator of phytophagous mites on apple trees, can be introduced into orchards by removing prunings from orchards with high populations and laying the prunings in the canopy of the trees [128].

In addition, fabric bands can be tied round tree trunks during the summer months and then transferred during the winter to areas with low predatory mite populations [129]. Fabric or cardboard bands provide refuge for a wide range of predators in apple and pear orchards and can be left in situ to provide year-round shelter [130,131]. Refuges are particularly beneficial in young orchards where the trees have not yet developed textured bark and crevices [132]. Cardboard and fabric bands can also be utilised by some pest insects, including codling moths, and act as an indicator of pest presence.

### 3.3. Avoiding Practices Harmful to Beneficial Insects and Conservation Biological Control

Conservation biological control aims to support beneficial organisms by enhancing and protecting their environment [133]. The addition of cover crops or floral resources can benefit substrate-dwelling insects by improving drainage and soil structure. Earwigs nest in burrows where eggs are laid and nymphs are reared by the parent female [134,135]. Kölliker [134] found that the disruption of the nest or death or exclusion of the female can cause the loss of the brood, reducing numbers of nymphs emerging in the spring. Although brood-adoption has been observed in some cases, with females adopting orphaned nymphs, the survival is greatly reduced and is not expected to be a frequent occurrence in the field [136]. Earwigs require good drainage to prevent the waterlogging of nests, but are also more prevalent in orchards with higher ground cover and minimal soil disturbance or compaction [89]. As females nest within the soil during winter and early spring [137], growers should avoid deep tillage and prevent soil compaction by minimising vehicular travel around orchards. It is possible that similar advice is appropriate for the main pollinators of apples, solitary ground nesting bees, but this requires further investigation. Although, in spring, it is often necessary to apply PPPs during flowering to protect blossoms from diseases, where possible, this should be kept to a minimum.

Ground-dwelling Coleoptera such as ground beetles (Carabidae) are polyphagous predators disrupted by tillage [138]. In cereals, rotary tillage reduced carabid activity by 52% in comparison to an untilled control [139], and in carrot fields the presence of vegetation cover, from no soil disturbance, promoted ground beetle presence [140]. Mechanical weeding may negatively affect soil invertebrates, including earwigs, and where possible should be kept to a minimum depth to reduce disturbance. Larger, more robust insects, such as ground beetles, seem more tolerant of this practice [141]. Mechanical weed control is less detrimental than herbicide applications to web spinning orchard spiders as weed re-generation is quicker with the former method [142].

The application of compost or mulches to suppress weeds in apple and pear orchards may be a more appropriate weed control practice. However, consideration of the source of mulch is needed, as mulch treatment can affect the viability of weed seeds. In apple, composted poultry manure applications to the base of trees resulted in weed suppression and an increase in predator population [143]. Benefits from compost application into the tree row include reductions in the populations of woolly apple aphid and spotted tentiform leafminer, *Phyllonorycter blancardella* (Fabricius) [143]. The timing of compost application also needs consideration. New, excessive shoot growth from high nitrogen inputs could promote aphid and pear sucker populations.

Fungicide applications to alleyways and tree spacings can negatively affect ground-nesting bees, which use the bare ground for their nests. Although the majority of fungicides will have no measurable impact on bees, some can cause periods of inactivity while the bees ‘recover’ [144], or impair orientation and nest recognition [145]. To ensure that ground-nesting bees are not exposed to harmful PPPs, applications should be made before nesting begins, prior to apple blossom [136].

In summary, less frequent mowing and careful consideration of the timing and type of PPP applied could foster natural enemies in orchards and improve pest control, ultimately leading to less reliance on insecticide applications.

### 3.4. Viruses

Species-specific viruses are used to target the codling moth and summer fruit tortrix, *Adoxophyes orana* (Fischer von Röslerstamm), which cause damage in apple and pear orchards. The granuloviruses belong to the baculovirus family (double-stranded DNA) [146]. They cause caterpillar mortality [147], and to date have had no reported impacts on non-target species. In a typical year, in a temperate climate, summer fruit tortrix and codling moth have their first generation between May and July and their partial second and full second generation between August and September, respectively, in the UK. Larvae pupate on the ground in leaf litter and soil, but also in bark crevasses or in splits in tree stakes and tree ties. The codling moth and summer fruit tortrix are resistant to many plant protection products [55,148,149,150,151], a major driving factor in the development of commercial viruses. The application of the viruses is targeted to coincide with egg hatching and larval feeding on the surface of fruit to ensure infection [152]. The timing of application is critical. For the codling moth, the first applications typically occur at 111–139 °C degree days [153], which for the UK is usually during May, but applications can also be timed by monitoring adult populations with species-specific pheromone traps [154,155]. Field trials employing viruses reduced larvae in the shoots by 97%, damage to fruit by 50–60%, and increased tortrix larval mortality by >81% [156]. There was a >77% reduction in fruit deep entry wounds when applied against codling moth [154]. The timing of pest generations can be predicted with models which use local temperature data to predict larval hatching. RIMPro software (Relative Infection Measure Pro) uses temperature, rainfall and humidity data and applies them to simulation models developed for apple and pear in Europe [157]. Codling moth and apple sawfly, *Hoplocampa testudinea* (Klug), egg hatch can also be predicted by RIMPro models [158,159]. Resistance to viruses is likely as individuals with low viral tolerance become removed from the population and offspring with a higher tolerance inherit resistance to the virus. This occurred in populations of codling moth in southeast France to the CpGV-M strain of granulovirus, which was used for 15 years. This barrier was overcome by developing new viral strains [160]. At the time of writing, there are no other viruses approved for use in the UK on pests in apple and pear, although several baculoviruses are currently being produced to control other global lepidopteran pests [161]. In addition, the development of viruses for the control of other key pests would greatly benefit growers by increasing control options that are species-specific.

### 3.5. Entomopathogenic Fungi and Nematodes

Entomopathogenic nematodes and fungi can be effective strategies pest control agents and are IPM and organic compatible [162]. Both are naturally occurring in the environment but are formulated to contain different species or stains for optimum efficacy. Generally, they have a broad host range with no impact on vertebrates and require specific environmental conditions to be effective.

Nematodes are commonly used for control of vine weevils, *Otiorhynchus sulcatus* (Fabricius) [163], and slugs [164]. Cross et al. [12] highlighted the potential of soil-applied nematodes for the control of pests which spend some of their lifecycle below ground, (e.g., weevils, tortrix moths, and codling moths). Apple sawfly is a common pest of dessert apple with the varieties Discovery and Worcester being highly susceptible. Controlling sawflies in organic orchards is challenging because the most effective PPPs are not available to organic production. Adult sawflies emerge before blossom and once the larvae have excited the apples in May, the rest of the lifecycle is spent belowground in a prepupal or pupal form [165]. Once applied to a substrate [166], nematodes locate a host by following CO_2_ trails [167]. Nematodes can also be applied to plant foliage. In field trials, where four foliar applications of *Steinernema carpocapsae* (Weiser) were applied to apple trees, secondary sawfly damage was reduced by 19% compared to an untreated control [168]. When applying nematodes as a foliar application, growers should be aware that in-field conditions, such as low humidity or high temperature, can make control variable [169].

Pear sawfly, *Hoplocampa brevis* (Klug), has a similar lifecycle to apple sawfly, spending several months in the soil. The pear sawfly can be controlled with the nematode, *Steinernema feltiae* (Filipjev), using both foliar and soil applications [170], but the optimum temperature for this nematode can limit its effectiveness, becoming inactive when the soil temperature is below 10 °C. There are currently very few reports of pear sawfly causing economic losses in the UK. Rising average temperatures during the summer could increase the incidence of this pest, as it is common in its native range of Asia and warmer European climates, such as Italy [171].

**Figure 2:**
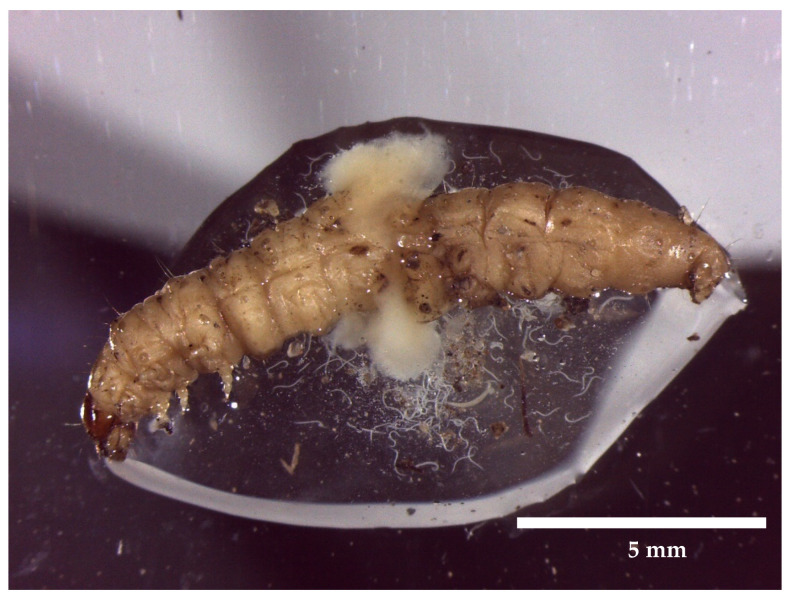
*Steinernema carpocaspsae* (Weiser) and *Steinernema feltiae* (Filipjev) nematodes released from codling moth larvae, *Cydia pomonella* (L.), within laboratory experiments.

In an unpublished study by Fountain et al., 2020, *S. carpocaspsae* and *S. feltiae* were applied to codling moth larvae in a series of laboratory tests (Figure 2). At 50 and 100% field rates, 62% and 100% mortality occurred in treated larvae, respectively. There is evidence to show that *S. carpocaspsae* and *S. feltiae* can be applied to reduce the survival of codling moth larvae as a foliar application [172] and woolly apple aphid as a spot treatment [173] in the field. However, foliar application of nematodes requires high moisture levels to ensure that nematodes do not desiccate and are able to reach the target host. Although there are several publications showing the efficacy of nematodes for pest control, they are rarely employed in orchards [162]. This may be because control is often difficult to quantify in a field setting or incorrect application timing or unfavourable conditions result in reduced efficacy.

By 2020, 750 species of entomopathogenic fungi (EPF) had been identified that infect a wide range of invertebrate hosts from virtually all orders of insects [174]. Almost all infect the host through the cuticle, and spores are picked up from brief contact with an inoculated surface [175]. As with many control options discussed in this review, uptake of EPF as a pest control tool in horticulture has been minimal [176]. This may be because EPF do not kill the pest instantly and the pest may continue to feed and reproduce for some time after infection. Generally, the processes of infection, once a host has come into contact with the EPF, can take between 7 and 14 days for symptoms and finally death to occur [176]. However, EPF spores are persistent in the environment, even when hosts are absent or when environmental conditions are unfavourable. Once hosts are available, EPF have the potential to repeatedly cycle within the host population providing-season long pest mortality [177]. Organic growing environments typically have higher diversity and abundance of EPF than conventional systems [178]. While there are reports of detrimental impacts of PPPs on EPF in laboratory trials [176], Clifton et al. [179] concluded that in the field, negative impacts of growing practices used in conventional production such as tillage and soil disturbance are more likely to reduce EPF abundance.

Rust mites (Eriophyidae) are a secondary pest in apple (*A. schlechtendali*) and pear (*E. pyri*) orchards. Apple rust mite can be controlled with the predatory mite *Typhlodromus pyri*; however, *T. pyri* is less common in pear trees, probably because the leaves have fewer trichomes (hairs) and offer the predator less protection. Both pest species are small (0.13–0.16 mm) and are wind dispersed [180]. Effective Eriophyidae population suppression has been achieved using several strains of EPF in other crops through foliar applications in laboratory and field trials [181]. To date, only one study has been published on EPF control in apple rust mites [182], and there are no reports for EPF efficacy on pear rust mites. Ninety-eight percent apple rust mite mortality, 6 days after the application of *Paecilomyces lilacinus* (Thom) to apple leaves, was observed in laboratory bioassays [182]. However, high humidity levels resulted in higher spore concentration than might be experienced in the field, indicating that applications after rainfall may be beneficial (as observed by Yagimuma [183]). Historically, EPF have more consistent efficacy when applied to target soil dwelling pests, due to the humidity required for sporulation [12]; nevertheless, their efficacy as foliar applications has greatly improved over the last 5 years. Advances in formulation [184], microencapsulation [185], application technology, and application setting (i.e., in unprotected cropping) [176] have enhanced the reliability and consistency of fungal-based insecticides.

### 3.6. Parasitoids

Parasitoids are insects of which the larval stages feed on, and eventually kill, an arthropod host [186]. Parasitic wasps (Hymenoptera) are the most widely known endoparasitoids. Female parasitoid wasps have an elongated ovipositor enabling them to lay eggs in the host. The release of parasitic wasps is a common strategy in soft-fruit production, typically in protected growing systems, but natural parasitism does occur in orchards. The review by Cross et al. [75], over 20 years ago, is still the most comprehensive report on the uses of European parasitoids to target pest insects of apples and pears. The authors highlighted that there is a wide range of parasitoids in orchards, although their impact on pest populations is probably minimal as individual species. However, multiple species may contribute to significant control in the absence of PPPs.

Apple sawfly is parasitized by the Ichneumonid, *Lathrolestes ensator* (Brauns). This wasp lays eggs during a two-week period, targeting the first and second larval instars [165]. This short window of opportunity can be disrupted by poor weather conditions. In addition, due to variation in flowering and fruit development time, varying rates of parasitism occur on different cultivars [187], depending on whether cultivar phenology is synchronized with that of the parasitoid. Rates of parasitism by *L. ensator* are affected by individual orchard and the management strategy used. Generally, parasitoid species richness is higher in organic orchards compared to conventional or IPM orchards [188] due to the detrimental impact of chemical PPP applications [75]. However, the occurrence of *L. enactor* can also be impacted in organic orchards by sulphur applications during parasitoid flight, and *L. enactor* is found more commonly in orchards with sandy soils [189,190].

Woolly apple aphids decrease plant health and can result in yield loss at high population levels. *Aphelinus mali* (Haldeman) is the main parasitoid wasp of the woolly apple aphid. Female wasps can oviposit 85 eggs within a lifetime. However, *A. mali* is temperature-limited, with slow reproduction rates below 25 °C, restricting its effectiveness in northern temperate climates [191]. Quarrell et al. [192] investigated the ability of *A. mali* to suppress woolly apple aphid populations aided by earwigs, which predate earlier in the season at cooler temperatures. They found that >14 earwigs per tree were required to suppress woolly apple aphids, and where this level was not met, >0.5 *A. mali* females per tree were required to prevent ‘severe’ infestation. They concluded that where these densities of natural enemies occurred in orchards, PPP may not be required to control woolly apple aphids.

Several weevil species are damaging in apple and pear orchards, including the pear blossom weevil *Anthonomus spilotus* Redtenbacher, apple bud weevil *Anthonomus pyri* Kollar, and apple blossom weevil *Anthonomus pomorum* (L.) [193]. While there are associated parasitoids, mainly from the Pteromalidae family (Hymenoptera), these are not effective for seasonal control, because the weevils have only one generation per year. In addition, due to the lengthy underground life stage of these weevils, the opportunity for parasitism to occur is time-restricted. However, *Centistes delusorius* (Foerster) hibernates within apple blossom weevil adults as a larva and pupation occurs in late spring, with adult parasitoids emerging to coincide with the emergence of weevil adults [194]. As discussed in previous sections, floral margins provide food (pollen and nectar) and shelter for parasitoids [195], which may increase their lifespan and rate of parasitism.

Codling and tortrix moths (primarily, summer fruit tortrix *A. orana*, and tree fruit tortrix *Archips podana* (Scopoli)) can be targeted by egg, larval, and pupal parasitoids, but attempts to employ these as augmented releases have been unsuccessful in Europe. This has been attributed to the high PPP input associated with control of these moths. As low populations of these pest species result in economic damage, growers typically employ PPP applications timed to coincide with adult moth flight, and hence egg hatch. Fruit damage by tortrix caterpillars is economically damaging at low pest population numbers. As parasitoids are sensitive to broad-spectrum PPP, the two methods are not complementary. However, in organic orchards, parasitoids (both native and introduced) have a positive effect on pest control [75]. Anecdotally, in unpublished field trials in the UK, 50% of the samples of tortrix larvae from chemically untreated plots were parasitised by various parasitoid species, but it is estimated that 10–20% parasitism is more typical [196]. In Swedish apple orchards, following the withdrawal of azinphosmethyl (a broad-spectrum product applied to control codling moth), population densities of *A. orana and A. podana* increased, but no increase in damage was observed [197]. Although several factors were thought to contribute to this response, the use of more selective PPP appears to have had a positive impact on predation and parasitism. Parasitoids are used very successfully in New Zealand to target codling moths, where augmented releases 50 years previously are now identified in crops where they were not recently released [198]. Before parasitoids such as *Ascogaster quadridentata* Wesmael can be released to control codling and tortrix moths, a consideration of which PPP are going to be employed needs to be taken into account.

The registration process for the release of non-native species for augmented biocontrol is often lengthy. As the introduction of alien species can severely disrupt native ecosystems, careful consideration is needed before they can be released [199]. In the event of an invasive pest species entering a region, there is often a lag between pest detection and parasitoid release, due to the approval process. However, researchers in New Zealand have pushed forward the approval process for the release of the Samurai wasp, *Trissolcus japonicus* (Ashmead), a specialist parasitoid of the brown marmorated stink bug (BMSB), *Halyomorpha halys* Stål, before it established in New Zealand. This invasive species, originally from Asia, has extended its range to include the United States of America and Europe, aided by its habit of ‘hitchhiking’ during aggregation. BMSB is a polyphagous pest of many crops, but also invades buildings during aggregation, causing an unpleasant odour [200]. By having a preemptive non-native parasitoid wasp approval in place in New Zealand, releases of the wasp can be performed immediately on the detection of BMSB living free in the environment. Researchers have also evaluated the possible impacts of this species on native stink bugs [201], and how it will interact with the environment [202].

### 3.7. Semiochemicals

Sex pheromones have been, to date, the most widely investigated and exploited semiochemicals for pest monitoring and control, although other pheromones, such as alarm, trail and aggregation pheromones, have been identified and characterised for many species [203]. The ability to synthetically produce semiochemicals has enabled their exploitation for pest monitoring and control because they are target-specific and have minimal impact on non-target species [204]. Once pheromones have been identified [205] and successfully synthesized, they can be employed in control strategies including mass trapping and mating disruption [206]. Monitoring using pheromones provides an accurate, localised approach and a species-specific detection method to make real-time crop protection decisions [207].

Pheromone monitoring traps are also a useful tool to aid growers in the detection of new, invasive species. They can be deployed in habitats known to be favourable to the pest, saving time and resources on physical searches. Apple and pear are two BMSB host plants and direct feeding damage to the fruits causes high yield losses if not controlled [208,209]. BMSB aggregate in human dwellings during the winter and the aggregation pheromone, which is produced to attract conspecifics, has been identified and synthesised for use in pheromone trapping. Pheromone traps are used to aid monitoring in countries that do not yet have the pest [210]. In West Virginia, a recently invaded area, trap catch thresholds, trap position, and resulting economic damage were investigated [211]. In apple orchards, two black pyramid traps baited with BMSB pheromone were deployed, at the edge and centre of each orchard and checked weekly. If an effective PPP was applied within a week of a capture of 1 to 10 BMSB individuals, there was a significant reduction in fruit damage compared to trap catches of 20 BMSB per week or an untreated control. Unfortunately, there are no organic products currently available to target BMSB. However, due to the severity of this pest, there is a pressing need to develop alternative, biological, and organically approved control products.

Species-specific aggregation pheromones combined with plant volatiles in mass traps can attract both insect pest sexes. This approach has been used for soft-fruit pests such as the strawberry blossom weevil *Anthonomus rubi* (Herbst), European tarnished plant bug *Lygus rugulipennis* Poppius [212,213,214,215], and raspberry beetle *Byturus tomentosus* De Geer [216]. The application of mass trapping in pome fruit is not common practice although it would be beneficial to develop this for insects with aggregation pheromones, e.g., weevils.

Pheromones can also be deployed without trapping devices for effective pest control. Codling moth is one of the most damaging insects in apple and pear crops, worldwide, and has insecticide resistant populations [217], which has driven the development of several organically approved control strategies. Mating disruption is an area-wide management practice that exploits the adult insects’ mate finding behaviour. This system works by releasing species-specific female sex pheromone over the treated area, preventing males from locating the females and subsequent mating [218]. First proposed in the 1960s [219], mating disruption use began commercially in the 1990s with varying levels of success [220]. Since then, mating disruption has evolved and has become extremely successful for several moth species; however, it has limitations and knowledge gaps [221,222,223]. For the codling moth, a dispenser is loaded with a synthetic formulation of the female sex pheromone. Dispensers can either be in the form of a device impregnated with the pheromone (passive), applied as a regular aerial spray, or via a timed-release aerosol (‘puffer’). The passive dispensers are distributed within the orchards at high densities, typically between 200 and 3000 units per hectare depending on manufacturer recommendations [224], and are labour-intensive to deploy and collect at the end of the season [225]. Aerosol dispensers can be timed to release pheromones at specific times to coincide with the female’s natural pheromone release, reflecting ‘calling’ behaviour, which for codling moth occurs at dusk. These aerosols are deployed at a much lower density per hectare, typically 2–4, depending on the manufacturer [224]. McGhee et al. [226] concluded that to provide the same atmospheric saturation as the passive mating disruption technique, five aerosol units per hectare are needed. Pheromones can ‘camouflage’ calling females, but also employed as false trail following, diverting the male moths away from females [226]. In addition, exposure to high quantities of sex pheromones can result in male olfactory receptors becoming non-functional, preventing the further detection of sex pheromones; synthetic or natural. Codling moth mating disruption development and implementation was covered in detail by Knight et al. [227]. This approach may also be combined with the sterile insect technique (see below).

In laboratory studies, conducted by Verheggen et al. [228], the presence of a synthetic aphid pheromone in cages containing prey resulted in an increase in foraging behaviour and oviposition by female hoverflies. In unpublished work by Fountain et al., 2020, several volatiles and blends were successful in attracting hoverflies, and other beneficials, including common green lacewings, *Chrysoperla carnea* (Stephens), into cropping areas. Methyl salicylate released from a range of plants under attack from herbivorous insects has been used to attract hoverflies and lacewings into apple orchards (Fountain unpublished), and was also found to be effective in attracting lacewings [229], ladybirds and *Orius* [230] into hop gardens.

Semiochemicals can also be deployed as repellents, deterring pests away from a crop or even disrupting behaviour [231]. These disrupting cues can be based on a range of volatiles including alarm pheromones and even repellent plants, inter-cropped within the orchard. The common green capsid, *Lygocoris pabulinus* (L.), has historically been an infrequent pest on apple and pear trees [213], although, along with other mirid species, it is expected to become more common in the future. Groot et al. [232] identified the female alarm pheromone of the common green capsid, and virgin females housed in a monitoring trap in combination with the alarm volatile caught only 1 male over a 30-day period, in comparison to the female alone treatment that caught 36 males. In the presence of hexyl butanoate, male common green capsids were less attracted to females, which would reduce mating success and subsequent population development. Similarly, research has identified and demonstrated the repellence of *L. rugulipennis* in conventionally and organically grown strawberry crops, with a reduction in mirid presence in the crop and a reduction in fruit damage of up to 80%. In a follow-on project, *L. pabulinus* was successfully repelled from commercial raspberry crops, reducing fruit and foliar damage (Fountain unpublished). To date, no studies have tested this strategy in apple or pear orchards for mirid control.

Aromatic plants emit volatiles that have the potential to attract and/or repel a wide range of pest species. Where ageratum *Ageratum houstonianum* Mill., French marigold *Tagetes patula* L., and summer savory *Satureja hortensis* L. were planted, there was a reduction in summer fruit tortrix moths within organic apple orchards [233]. In these orchards, there was also an increase in parasitic wasps and diverse natural enemies of other pest species. French marigold, agreatum and basil *Ocimum basilicum* L. also reduced spirea aphid (*Aphis spiraecola* Patch) infestation on apple by 35%, 29% and 38%, respectively, in comparison to an untreated control. These plants act as a deterrent to the pest and an attractant to their parasitoids [234] and predators. Similarly, coriander *Coriandrum sativum* L. promoted lacewing oviposition in strawberry tunnels [235].

Apple blossom weevil is one of the most damaging pests in organic apple and can attack pear trees [236]. Synthetic volatiles of the walnut tree *Juglans regia* L. had a deterrent effect on apple blossom weevil in laboratory studies [237], but this research was not extended into pest control options. The pear blossom weevil, pear bud weevil *Anthonomus spilotus* Redtenbacher and apple bud weevil are also minor pests of pear trees [193]. Pheromones have been identified for *Anthonomus* pests on other crops, e.g., cotton boll weevil (*Anthonomus grandis* Boheman) [238], pepper weevil (*Anthonomus eugenii* Cano) [239], and strawberry blossom weevil (*Anthonomus rubi* Herbst) [240], indicating that volatile communication and detection is well preserved in this family. For this reason, it would be beneficial to identify the pheromones of orchard weevils to better monitor and exploit pheromones for control.

Semiochemicals used to attract (‘pull’) pests away from crops can be combined with repellent volatiles to ‘push’ the pest away from the area in a push–pull approach, which could exploit synthetic or natural volatiles. Semiochemicals can also be employed to pull natural enemies into the vicinity and target the pest [231,241]. This system is used effectively by subsistence farmers in Africa, the first area to employ this method, to control stem borer species (Lepidoptera) in maize [242]. There are several reports of the use of this system in vegetable and field crops [243], but its uses in fruit crops are limited. The implementation of push–pull approaches in apple and pear trees requires additional research. There are semiochemicals associated with several apple and pear pest (e.g., mirids, midges, tortrix, etc.) species and, hence, this represents an opportunity to exploit pheromones and plant volatiles in push–pull approaches in orchards.

## 4. Physical Control

### 4.1. Netting and Barriers

The netting of fruit trees has become common practice for stone fruits over recent years, especially with the introduction of spotted wing drosophila, *Drosophila suzukii* (Matsumura)*,* in cherries [244]. The netting or mesh is normally erected prior to pest occurrence in the orchards and physically prevents the pest from reaching the developing fruit. Netting apple and pear trees has historically been used to protect fruit from damage from environmental conditions such as sunburn, hail damage and high winds [245], but is infrequently used for pest control. In pear and apple trees, many lepidopteran, sawfly and weevil pests spend a proportion of their life-cycle within the soil, meaning trees would need to be netted with minimal or no soil accessibility or as individual rows rather than whole-orchard netting [246]. To do this, nets need to encase the canopy and be closed around the main trunk to be effective. In France, this approach has been successfully used within field trials to reduce fruit damage by codling moth by 91% in comparison to an un-netted control [247]. It is speculated that the reduction occurs via disrupting mating (by preventing moths from flying over the canopy to find mates) rather than suppressing oviposition. Fruit damage from mirids and birds was also reduced; however, woolly and rosy apple aphid species can increase in prevalence [246], presumably because an insect-excluding mesh creates a microclimate beneficial to aphids and/or prevents their main natural enemies from reaching trees (e.g., earwigs, hoverflies, ladybirds, etc.). In continental Europe, netting has been successfully used to prevent BMSB damage to apples to levels lower than insecticide treated plots. These authors also state that predator and parasitoid numbers were not reduced using this technique [248]. BMSB has not yet been confirmed as having a breeding population in the UK and is classed as migratory until such a time as juvenile life stages are found [14].

### 4.2. Waste Removal

The life cycle of some key pear and apple pests has a soil phase. Weevil, sawfly, midge, and lepidopteran pests can pupate in the soil beneath trees after migrating from the dropped fruitlets and foliage to the soil. For example, apple fruit weevil females sever the petiole of developing fruitlets once an egg has been laid. The larvae then access the soil for pupation. In soft- and stone-fruit, it has become common practice to remove all waste fruit from the cropping area, including the ground surface, and then treat the fruit to prevent re-infestation of spotted wing drosophila [249,250]. Currently, this has not been adopted in pome fruit management, presumably due to the high labour input needed to implement it successfully.

In some cases, growers have combined apple and livestock farming and used either sheep or pigs to graze on dropped fruit. With sheep grazing, there have been reports of increased levels of N, C, and P [251] (see Section 2.1 on soil health and properties) which can be beneficial or detrimental depending on cultivar. There are varying levels of success reported, but overall reductions in codling moth and tortrix damage the following season have been observed; pigs typically remove between 90 and 100% of dropped fruit [252,253]. Buehrer and Grieshop [252] identified a significant reduction in oriental fruit moth larvae in dropped fruit and significantly reduced codling moth and oriental fruit moth damage to fruit the following year in pig-grazed orchards, compared to untreated controls. There are many associated costs with implementing livestock grazing in orchards including fencing, animal husbandry, and licensing. Livestock can cause extensive soil disturbance, which could have impacts on ground-nesting/dwelling organisms. The choice of livestock breed and age should be carefully considered to prevent tree damage (more detail and case studies were provided by Grieshop [254]).

### 4.3. Particle Films

Particle films are mineral in composition and coat the target crop in a barrier which disrupts insect behaviour and protects fruit from damage [255]. Most films consist of kaolin, a white clay that has high reflectance and can be easily washed from fruit prior to sale. Kaolin films have been used successfully for the season-long control of olive fruit flies compared to an insecticide-treated grove, which was only protected while the last spray persisted [256]. For boll weevil, cotton plants treated with kaolin yielded 2.4 and 1.4 times more cotton than an untreated control and cotton treated with an insecticide, respectively [257]. The application of kaolin to control pear sucker reduced nymph density, which resulted in season-long suppression and an increase in pear yield [258]. Pear sucker prevalence was reduced by 75% in trees treated with kaolin in an unpublished study by Fountain et al., 2019, and has been suggested as a pre-bud burst treatment to control early season suckers. In warmer climates, the use of particle films reduced leaf-roller damage to leaves and heat stress in apple [259]. In the Netherlands, kaolin was reduced several key pest species including apple blossom weevil numbers, by 51% [260]. However, a disruption of earwigs and parasitoids was also observed and woolly apple aphid and rosy apple aphid incidence increased in the treated plots [260]. It would be beneficial to further investigate the timing and application methods of particle films for improved pest control whilst preserving natural enemies.

Physical pest control can be time-consuming and costly to initially implement in apple and pear orchards. However, physical barriers generally reduce pest pressure by preventing pest migration on to orchard trees, removing dependence on other control approaches. For insect exclusion netting, the initial cost of purchasing and deploying the materials appears high (396 EUR/ha including material, labour and machinery [244]), but the life expectancy can be 10–15 years if well maintained. It is likely that physical approaches to pest management may be more suitable for smaller orchards in which smaller machinery is used due to the hinderance enclosure netting has on orchard access. The implementation of livestock on some farms is not feasible due to the many other requirements. However, for existing livestock farmers this may be a low-cost approach to waste disposal and soil improvement.

## 5. Conclusions

While there are many organic options available to control pests in apple and pear orchards, several methods need to be combined for suppression below economic thresholds. Whereas a broad-spectrum insecticide may potentially eliminate many pests with one application, organic practices generally require several, accurately timed strategies and the integration of several control methods. For some control options, there may be an increase in labour requirements in installation, monitoring, and regular deployment. However, through employing these techniques, growers will build more resilient and sustainable control strategies year-on-year into the long term, rather than short-term fixes with faster-acting PPPs. To achieve this, more regular and accurate monitoring with a greater understanding of pest/natural enemy lifecycle and biology is required, coupled with information on the appropriate timing and environmental conditions required for effective pest control. There appears to be a lack of uptake from growers for some of the more effective methods, but this may be due to the availability of other easy and low-cost options. It is likely that uptake of the control methods has been prevented by the higher costs associated with many of the strategies. Labour costs associated with implementing many of the outlined approaches are also prohibitive. With the current labour shortages in horticulture, some of the methods discussed may not be possible. However, many of the suggested approaches require very little skill and some are not time-restricted within the season (i.e., deploying earwig refuges), and so could be reserved for quieter periods in the year.

From discussions with apple and pear growers, other than the cost, it is the speed of pest kill, high associated risk to fruit, and lack of demonstrable results on farms that deter confidence and uptake, particularly approaches such as EPF and nematodes. Enhancing environments for biological control, e.g., conservation biological control, can take years to fully establish. However, it is promising that there are many organic pest control options available for apple and pear growers and that there are pioneering growers willing to test and demonstrate implementation and efficacy. With expected changes in maximum residue limits for PPP and restrictions in the number of detected active ingredients (residues) in Europe, growers need to adopt alternative approaches to pest control.

This review has highlighted current and future approaches which are available to commercial organic and IPM orchards. We have also highlighted areas where there are gaps in knowledge that could be researched and further exploited for future pest control options. Although the foundations for future research are well-established, strategic funding is needed to fully explore, integrate, and implement new strategies under different orchard scenarios. Finally, growers need evidence of efficacy in field conditions and added incentives to employ methods which are often costly and impact net income.

## Figures and Tables

**Table 1:** Data collected by the UK Office for National Statistics on behalf of the Department for Environment Food and Rural Affairs (Defra) on apple and pear production values projected for 2020, based on 2019 data (accessed on 16 August 2021).

	2020 Provisional Data		
	Area Grown (Hectares)	Yield (Thousand Tonnes)	Value (£ Million)
Dessert Apples	6372	200.7	158.1
Culinary Apples	2473	92.2	81.2
Cider Apples and Perry Pears	6700	154.1	27.6
Dessert Pears	1470	25	19.9

## Data Availability

Not applicable.
